# Supplementary figures and images for: From data strategy to implementation to advance cancer research and cancer care: A French comprehensive cancer center experience
Source: PLOS Digit Health. 2023 Dec 19;2(12):e0000415. doi: 10.1371/journal.pdig.0000415 (PMC10729983; doi:10.1371/journal.pdig.0000415)

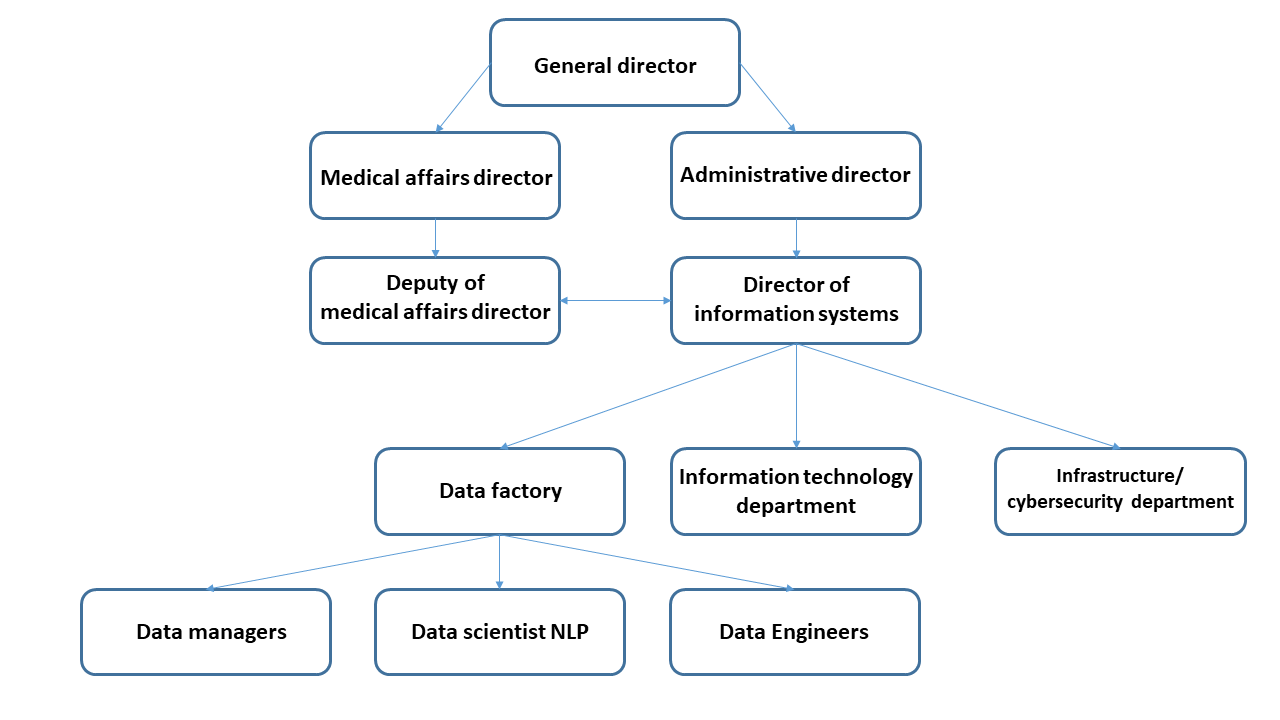

Supplement: S1 Fig — (TIF) [file pdig.0000415.s001.tif]

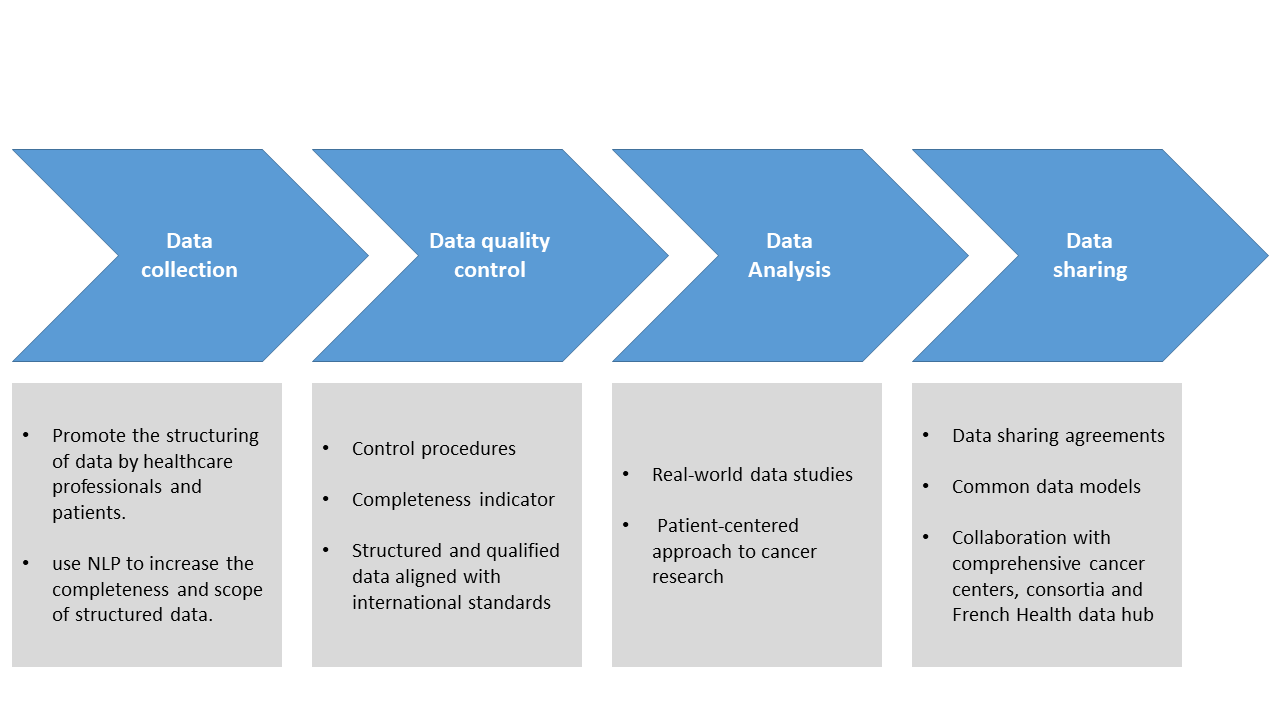

Supplement: S2 Fig — (TIF) [file pdig.0000415.s002.tif]

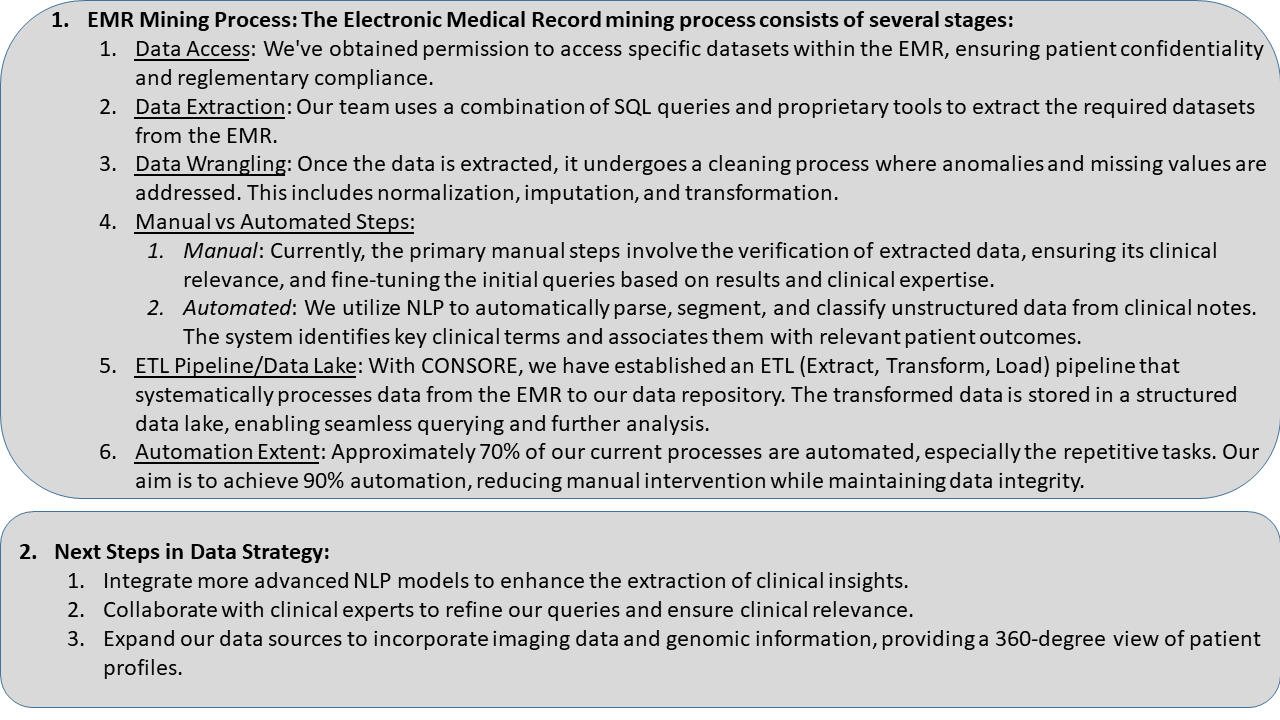

Supplement: S3 Fig — (TIF) [file pdig.0000415.s003.tif]
