# Supplementary material for: From data strategy to implementation to advance cancer research and cancer care: A French comprehensive cancer center experience
Source: PLOS Digit Health. 2023 Dec 19;2(12):e0000415. doi: 10.1371/journal.pdig.0000415 (PMC10729983; doi:10.1371/journal.pdig.0000415)
Supplement: Attachment — Submitted filename: Authors response letter v1.docx [file pdig.0000415.s004.docx]

Dear Reviewers

We would like to thank you once again for considering our paper for publication in Plos One Digital Health (Manuscript ID: PDIG-D-23-00112). We are pleased to send you a revised manuscript.

We would like to thank the 2 reviewers for their pertinent comments and suggestions that helped us to improve the clarity and consistency of our manuscript. Please see below our point-by-point response. The modifications proposed below in response to the reviewers’ comments have been incorporated in the revised manuscript and appear as « track changes ».

We hope that this new version of our manuscript will respond to all your requests and be further considered for publication in Plos One Digital Health.

On behalf of all authors, I look forward to reading from you.

Best regards,

**REVIEWER #1**

Dear authors, let me first take the opportunity to thank you for contribution to a topic in a rapidly evolving field that urgently needs to be streamlined regarding standardized strategies for processing data.

Research published in PLOS Digital Health should fulfill each of the following criteria:

1. Originality

2. High importance and broad interest to the community of researchers, engineers and clinicians working in the field of digital health

3. High methodological rigor and ethical standards

4. Substantial evidence for its conclusions

5. Clearly outlined utility and accessibility for the broader community

6. Follow appropriate standards and practice of open science

I therefore want to outline, that to fulfill all criteria, especially to add value to the scientific community, your manuscript needs to be supplemented and revised from my point of view. A major issue is that the manuscript is too superficial in some segments. I sometimes find it hard to recognize what qualifies your currently described work as original research as it is a description of your strategy, enriched by very few preliminary data gained from your specific pipeline at CLB. Nevertheless, I believe that with major revisions it should be able to improve this manuscript so it will be adding value to the scientific community.

Thank you very much for your thoughtful feedback on our manuscript. We greatly appreciate the time and effort you've invested in reviewing our work.

1. In your Introduction you criticize the rather too theoretical approach of scientific cancer societies and emphasize your practical approach with concrete examples. However, on the main elements your manuscript must face the same reproach.

We thank Reviewer 1 for their critical review and hope that the detailed answers and additional examples provided will meet expectations. We also propose to add this sentence in the introduction part : «*This theoretical and generalist approach is essential because it must be shared, but its practical application can most often only be developed at the institutional level.* »

2. In Material & Methods you summarize the historical development of the EMG at CLB. I suggest you add a graphical overview of the development over time to summarize your text. From such an overview it would become clear that the pace of development is rapidly increasing. Try to be as informative as possible when describing EMR elements regarding data structure, interoperability, and data sources as well as data providers. In addition, this figure would be of benefit for describing your vision of an idealized data lake.

Indeed, for greater clarity and easier reading of this paragraph, we suggest that you add figure 1 which shows all the computer software used, specifying their years of installation and whether they were developed in internally by our IT department or purchased from an external service provider.

« *Figure 1 : Applications patient centered* »

3. Moreover, a second figure to visualize the information system directorate and their respective interfaces would be helpful. Both figures together allow for a concrete glimpse of work in progress at CLB and can thus be of guidance for other centers that are struggling with the same task.

We suggest that you add supplementary figure 1 which allows you to present the organization chart of the different branches of the IT department as well as the links with the general director of centre Leon Berard and the medical community.

4. As for the results section, please prepare a figure for your 4-axis strategy that includes a preview of the results and gives the precise measures, your data strategy includes. Furthermore, it is unclear to which extent the EMR can be mined so far, as you do not precisely describe the process of mining, extracting and data wrangling. How much of these steps can be attributed to manual or automated steps, e.g. with NLP? Is there an ETL pipeline or a data lake? It would be helpful to know to which extent pipelines are automized so far and what your next steps are to further develop your data strategy.

Thank you for your valuable feedback. Let me clarify our 4-axis strategy and our processes related to data mining and wrangling from the EMR.

A. **4-Axis Strategy supplementary Figure 2 :** This figure schematizes the 4 main axes of our strategy, taking up the main points of each axis.
B. **EMR Mining Process**: The Electronic Medical Record mining process consists of several stages:
C. **Data Access**: We've obtained permission to access specific datasets within the EMR, ensuring patient confidentiality and reglementary compliance.
D. **Data Extraction**: Our team uses a combination of SQL queries and proprietary tools to extract the required datasets from the EMR.
E. **Data Wrangling**: Once the data is extracted, it undergoes a cleaning process where anomalies and missing values are addressed. This includes normalization, imputation, and transformation.
F. **Manual vs Automated Steps**:
G. **Manual**: Currently, the primary manual steps involve the verification of extracted data, ensuring its clinical relevance, and fine-tuning the initial queries based on results and clinical expertise.
H. **Automated**: We utilize NLP to automatically parse, segment, and classify unstructured data from clinical notes. The system identifies key clinical terms and associates them with relevant patient outcomes.
I. **ETL Pipeline/Data Lake**: With CONSORE, we have established an ETL (Extract, Transform, Load) pipeline that systematically processes data from the EMR to our data repository. The transformed data is stored in a structured data lake, enabling seamless querying and further analysis.
J. **Automation Extent**: Approximately 75% of our current processes are automated, especially the repetitive tasks. Our aim is to achieve 90% automation, reducing manual intervention while maintaining data integrity.
K. **Next Steps in Data Strategy**: We are planning to:

- Integrate more advanced NLP models to enhance the extraction of clinical insights.
- Collaborate with clinical experts to refine our queries and ensure clinical relevance.
- Expand our data sources to incorporate imaging data and genomic information, providing a 360-degree view of patient profiles.

We propose to add this sentence : « *Our EMR mining process involves stages like obtaining data access, using SQL and tools for data extraction, and then refining the data, which includes both manual verification and automated processing via NLP. With CONSORE we have implemented an ETL (Extract, Transform, Load) pipeline that feeds into a structured data lake. Our next steps focus on enhancing NLP capabilities, collaborating with clinical experts, and expanding data sources (incorporing imaging data and genomic information) for a holistic patient view.* »

5. For the patients-section, can you state if the characteristics summarized in Table 1 are raw extracted data or if they have been modified. First, the current table is only partially suited to gather further insights for a researcher: For the cancer type, metastatic, de novo metastatic and localized disease should be assigned to solid cancer as subsections. Moreover, cancer location is an incorrect label as you are describing the respective cancer entity. Cancer laterality is only relevant to distinct entities, such as colorectal or breast cancer but not for the overall subset. Cancer treatments as a summary is interesting but would be even more insightful, if therapeutic modalities were assigned to each cancer entity. The summary should focus on the three main pillars: systemic, surgical and radiotherapy. With regard to future trends, you should think about adding a fourth pillar, i.e. cellular therapies as well. These include CAR-T-cell therapy, bispecific antibodies and allogenous stem cell transplants. As a next you could aim to have this arranged over time.

We fully agree with the comment of reviewer 1. Table 1 is only intended to provide a very approximate inventory of the number of cases and in no way to present a precise description of all cases of cancers and of their therapeutic strategies. One can also count the number of patients treated with immune checkpoint inhibitors or car-T cells, but we think that would risk losing readers with too many numbers.

Additionally and in response to Reviewer 1's comment, we propose to add in Table 1: « *exclusively solid cancers* » for metastatic disease and de novo metastatic disease but also « *Unspecified/Not applicable* » concerning cancer laterality.

6. In conclusion, I suggest that you define a meaningful summary table with the oncology team and try to arrange your ETL pipeline accordingly. If, what you have summarized in the table is the raw output, it should be arranged differently, as the way the data is presented now, it is not informative.

Being a medical oncologist myself, I agree with Reviewer 1 that Table 1 and Figures 2, 3 and 4 do not provide medical information. Indeed, the only objective of the presentation of these numbers and these graphs is to demonstrate that it is possible to easily extract raw data from the whole corpus of patients and in no case to present a statistical analysis or assess a particular neoplastic situation. In order to clearly specify this objective, we suggest that you add this sentence to the text presenting this data :  « *These raw data from the entire corpus of patients as well as the following graphs aim to demonstrate the technical feasibility of easily obtaining data but in no way to carry out a statistical evaluation of a neoplastic situation or a therapeutic strategy.* »

7. For the acquisition of side effects, you describe to have implemented a software. Please be specific and describe the way the software works and if it is a commercial software or self-made and if or how it was implemented into your pipeline.

In order to precisely answer this question, we propose to add in this paragraph that the software was homemade (« *internally developed* ») and to add this sentence : « *These data are collected manually by medical doctors, coordinating nurses/ Advanced practice nurse or clinical research associates.* »

8. For the data qualification section, you compared data extracted via CONSORE with the validated data from the PROFILER trial. Please be more specific, describe the trial in a few sentences.

We propose to add this sentence :« *The primary goal of PROFILER trial was to analyze molecular profiles of advanced cancer patients and to recommend therapies based on genetic mutations.* »

9. What you describe in data analysis strategies, data sharing strategies, general data protection regulation (GDPR) and data interoperability and the FHIR and OMOP standards is not part of the results section but rather reads like part of the material & methods section.

As suggested by Reviewer 1, we therefore propose to move this paragraph to the Materials and Methods section.

10. In your discussion, you correctly state the current sparsity of your data but in my view, you lack a future vision of how your data strategy can be developed further in a stepwise fashion to comprehensively characterize cancer patients at CLB. Is it necessary to digitize to make WSI available? Or is it of more value to have the results of molecular diagnostics such as NGS at hand to combine them with clinical data to get meaningful information? Finally, I was confused to read about three main axes in the discussion as you have mentioned four main axes at the beginning of the results section.

Even if these different projects are all important and must be developed in parallel, it is of course important to take into account in our strategy that their timetable will be different. The implementation of a digital pathology service will require a lot of time and support (human and computer change), while one can imagine that the alignment of biological results on the LOINC classification will not disturb the habits of doctors and that better integration of NGS results could help clinicians in their daily practice. We suggest adding these following 2 sentences in the mentioned paragraph : «*The integration of NGS results with clinical data offers a more comprehensive understanding, allowing for a personalized treatment approach.*  »… « *While Whole Slide Imaging (WSI) through digitization can provide an immense wealth of morphological details, its true potential is amplified when combined with other diagnostic methods. Thus, making WSI available is a step towards a holistic patient profile, but not an end in itself.* »

We thank the reviewer for pointing out this oversight. We propose this correction : « *In summary, data collection, quality control, analysis, and sharing strategies are essential to improving patient outcomes and advancing cancer research* ».

11. To conclude, there a several mistakes in grammar in spelling which must be corrected. Please find enclosed a selection:

Line 66): Cancer is … Line 71/72): … a variety of … Line 114): The project uses … Line 66): Cancer is …

Thank you for this feedback, we have tried to correct spelling and grammar errors.

**Reviewer #2:**

The authors present on an approach to data collection, analysis, and sharing to enhance their understanding of cancer and improve patient outcomes. Leveraging advanced data analytics techniques and developing effective data sharing policies can help cancer centers effectively harness the power of data to drive progress in cancer research.

We thank Reviewer 2 for their interest in this topic and we share his opinion.

Major:

1. Section Data Qualification: Is the quality control a procedure only performed once? Or are there workflows in place or plans to conduct routinely data quality assessments and reports? Are there standards used to follow such routine workflows?

We thanks reviewer2 for this question. Quality control is certainly a time-consuming but very important element. To date, the only quality control carried out systematically is that of the control of the data obtained by CONSORE during each version upgrade, i.e. approximately twice a year. Then, quality control mainly concerns the data extracted for research projects. In the short term, our desire is to set up a precise organization (probably around the organization of multidisciplinary consultation meetings) making it possible to know for each "tumor file" the author and the date of validation.

We suggest adding this sentence at the end of the paragraph: « *Quality control, currently done twice a year by CONSORE during version upgrades, focuses on data for research projects, with plans to establish a structured process to track authorship and validation dates for each tumor file.* »

2. Data Analysis strategies: Please provide references for you statements on read world data studies in this paragraph(page 12).

We fully agree with this request from reviewer 2 and to bring more precision to this paragraph, we propose to add these 3 following references :

- *Tang M, Pearson SA, Simes RJ, Chua BH. Harnessing Real-World Evidence to Advance Cancer Research. Curr Oncol. 2023 Feb 2;30(2):1844-1859. doi: 10.3390/curroncol30020143. PMID: 36826104*
- *Prince RM, Atenafu EG, Krzyzanowska MK. Hospitalizations During Systemic Therapy for Metastatic Lung Cancer: A Systematic Review of Real World vs Clinical Trial Outcomes. JAMA Oncol. 2015 Dec;1(9):1333-9. doi: 10.1001/jamaoncol.2015.3440. PMID: 26378774*
- *Ortega-Franco A, Hodgson C, Raja H, Carter M, Lindsay C, Hughes S, Cove-Smith L, Taylor P, Summers Y, Blackhall F, Califano R. Real-World Data on Pembrolizumab for Pretreated Non-Small-Cell Lung Cancer: Clinical Outcome and Relevance of the Lung Immune Prognostic Index. Target Oncol. 2022 Jul;17(4):453-465. doi: 10.1007/s11523-022-00889-8. Epub 2022 Jul 4. PMID: 35781861*

3. Section Data sharing strategies: Please add results to this section. Maybe you can explain (diagram?) your workflows in how you were able to successfully share your data with outside institutions?

Indeed, we propose to add the following sentence with 3 references in order to give some examples of projects : «*Many multicenter research projects are underway with this national platform »*

*29. DEEPSARC. Available online : https://www.health-data-hub.fr/partenariats/deepsarc. Accessed on August 2023*

*30. Health data hub. Available online : https://www.health-data-hub.fr/actualites/le-centre-leon-berard-inria-et-sanofi-sassocient-avec-le-health-data-hub-pour-mieux. Accessed on August 2023*

*31. UNIBASE. Available online https://www.centreleonberard.fr/institution/actualites/2-projets-de-recherche-du-centre-leon-berard-laureats-dunibase. Accessed on August 2023*

Unfortunately, we don't have any quantified results to give nor a very precise workflow because each research project has specific technical and regulatory requirements.

4. Section GPDR: Are the mentioned two levels of patient information available? Can they be linked or referenced here? Was there feedback from patients or researchers on the usability of this information?

Thank you for this remark. We suggest that you add this reference, which is an example of general information sent to all our patients (only in French) :	[*https://www.centreleonberard.fr/patient-proche/vous-accompagner/vos-droits/vos-donnees-de-sante*](https://www.centreleonberard.fr/patient-proche/vous-accompagner/vos-droits/vos-donnees-de-sante)

Unfortunately, we do not have an example of an individual information note online (most often sent by We have not evaluated the satisfaction of our patients and our researchers on this subject, but it is an excellent idea. Thank you.

5. Section Interoperability: Are the referenced papers results of the project? If not, can you focus this section on your results or describe the planned results of the project abit more in depth?

Indeed, this paragraph mainly explains a short-term future objective which is the alignment of our data model on these 2 international references. Unfortunately, we don't have any results to present yet. We conclude this chapter with this sentence for clarity :

« *Given the work already undertaken on the OSIRIS data model, our ultimate goal is to align this model with OMOP and FHIR to facilitate data sharing during international projects.* »

Minor:

6. Please check if section „Data Qualification“ should be renamed to „Data quality control“ 7. Please check if capitalization of letters in title and sections are compliant with the journal guideliens

Thank you for this last comment, we propose to use the term «Data quality control » and we will see with the editor if the graphic style is correct.
