# Supplementary material for: From data strategy to implementation to advance cancer research and cancer care: A French comprehensive cancer center experience
Source: PLOS Digit Health. 2023 Dec 19;2(12):e0000415. doi: 10.1371/journal.pdig.0000415 (PMC10729983; doi:10.1371/journal.pdig.0000415)
Supplement: Attachment — Submitted filename: Author response letter v2.docx [file pdig.0000415.s005.docx]

Dear Editor and Reviewers

We would like to thank you once again for considering our paper for publication in Plos One Digital Health (Manuscript ID: PDIG-D-23-00112). We are pleased to send you a second revised manuscript.

We would like to thank again the 2 reviewers for their pertinent comments and suggestions that helped us to improve the clarity and consistency of our manuscript. Please see below our point-by-point response. The modifications proposed below in response to the reviewers’ comments have been incorporated in the revised manuscript and appear as « track changes ».

We hope that this new version of our manuscript will respond to all your requests and be further considered for publication in Plos One Digital Health.

On behalf of all authors, I look forward to reading from you.

Best regards,

Reviewer #1: Dear Editor and Authors,

thank you for having me review the revisions for the manuscript entitled "From Data Strategy to Implementation to advance cancer research and cancer care: A French comprehensive cancer center Experience". I will briefly comment on my points previously raised:

First of all, I would like to ask the supplemental figures to be added to the manuscript, as I have otherwise no way to evaluate them.

1) This comment was indicated to ask for more detail in the manuscript. This request was complied with in part throughout the revisions.

We thank Reviewer 1 and hope that the changes made in this new version will bring even more precision to this manuscript.

2) Figure 1 has been composed and helps to get a quick overview of the many measures utilized. However it still lacks a good overview and more detail (What is a "Tumoral resources center"; what does a "Request for medical advice mean?" Is it a simple consultation request form or something more sophisticated?. A figure description is missing. Moreover, the colors chosen (pink) make it harder to read. I moreover suggest to add some kind of chronological order. The formatting is mixed within the figure. Finally I suggest changing the title "Applications patient centered" as I am struggling to see how it is related to the contents.

We would like to thank Reviewer 1 for this very important comment confirming the need to provide more information in Figure 1. In order to further clarify Figure 1 in more detail, we propose adding the following paragraph:

« *The Biological Resources Center and the information system allow us to specify, for each patient, all quantities of biological materials (tumors, blood, plasma, etc.) that are still available. The medical review form is a simple digital form that nevertheless enables the collection of digital data (dermatology photos, indications for oncogenetic investigations, or specific symptoms for gynecological consultation requests.)*'".

As for the title, we suggest keeping this title «*Patient-centered Applications* » , as it encompasses all applications revolving around the patient record, ultimately providing valuable information for patient care or research projects.

Finally, we changed the color pink.

3) I would like to see Supplementary Fig 1

We sincerely apologize that reviewer 1 was unable to evaluate Supplementary Figure 1 which was well integrated during submission. We will check with the editor that there have been no technical problems.

4) I would like to see Supplementary Fig 2 and I appreciate your detailed description of the EMR mining process. However, I am convinced the reader benefits from the details as well, which is why I suggest integrating them to the manuscript of within another figure

We will check with the publisher that there have been no technical problems and we sincerely apologize that reviewer 1 was unable to evaluate Supplementary Figure 2. We propose to integrate our EMR Mining Process and our next steps in Supplementary figure 3.

5) This comment has been sufficiently addressed.

6) This comment has been partly addressed. However, if the sole purpose is to demonstrate feasibility, reducing the number of figures and moving these additional figures to the supplement adds to the clarity and conciseness of the manuscript.

We thank Reviewer 1 for this comment. We agree that Table 1 and Figures 2, 3, and 4 do not provide medical information but rather offer an insight into the volume of cancer patients treated at our hospital, making it easy for our readers to compare with their own institutions or partner hospitals.

7) This comment has been sufficiently addressed.

8) This comment has been sufficiently addressed.

9) This comment has been sufficiently addressed.

10) This comment has been sufficiently addressed.

11) Please once more check for errors in grammar an spelling, as a well-written manuscript is easier to follow: "Cancer is a disease ..." etc.

We apologize for this typo and hope that we have corrected them all.

Reviewer #2: (No Response)
